# Correction: Dietary restriction improves repopulation but impairs lymphoid differentiation capacity of hematopoietic stem cells in early aging

**DOI:** 10.1084/jem.2015110012042020C

**Published:** 2020-12-17

**Authors:** Duozhuang Tang, Si Tao, Zhiyang Chen, Ievgen Oleksandrovich Koliesnik, Philip Gerald Calmes, Verena Hoerr, Bing Han, Nadja Gebert, Martin Zörnig, Bettina Löffler, Yohei Morita, Karl Lenhard Rudolph

The authors regret that in the original version of Fig. 1 B, the legend should have stated *n* = 3–5 mice per group, not 4–5 mice per group. In addition, the P value for the 1-mo CD150^lo^ time point should have been **, P < 0.01, not ****, P < 0.0001. The corrected figure and legend are shown here. The authors also clarify that the P values in Fig. 2, E–G; Fig. 5, A–C and F–I; Fig. 6, F–J; Fig. 7, D–H; Fig. 8; and Fig. 9, A–C, E–K, and M–P depict results derived from the unpaired Student’s *t* test. The one-way ANOVA indicated in the legends was employed to confirm that the depicted differences were significant in multi-comparison testing. The errors in Fig. 1 appear in print and in PDFs downloaded before December 3, 2020.

**Figure 1.**
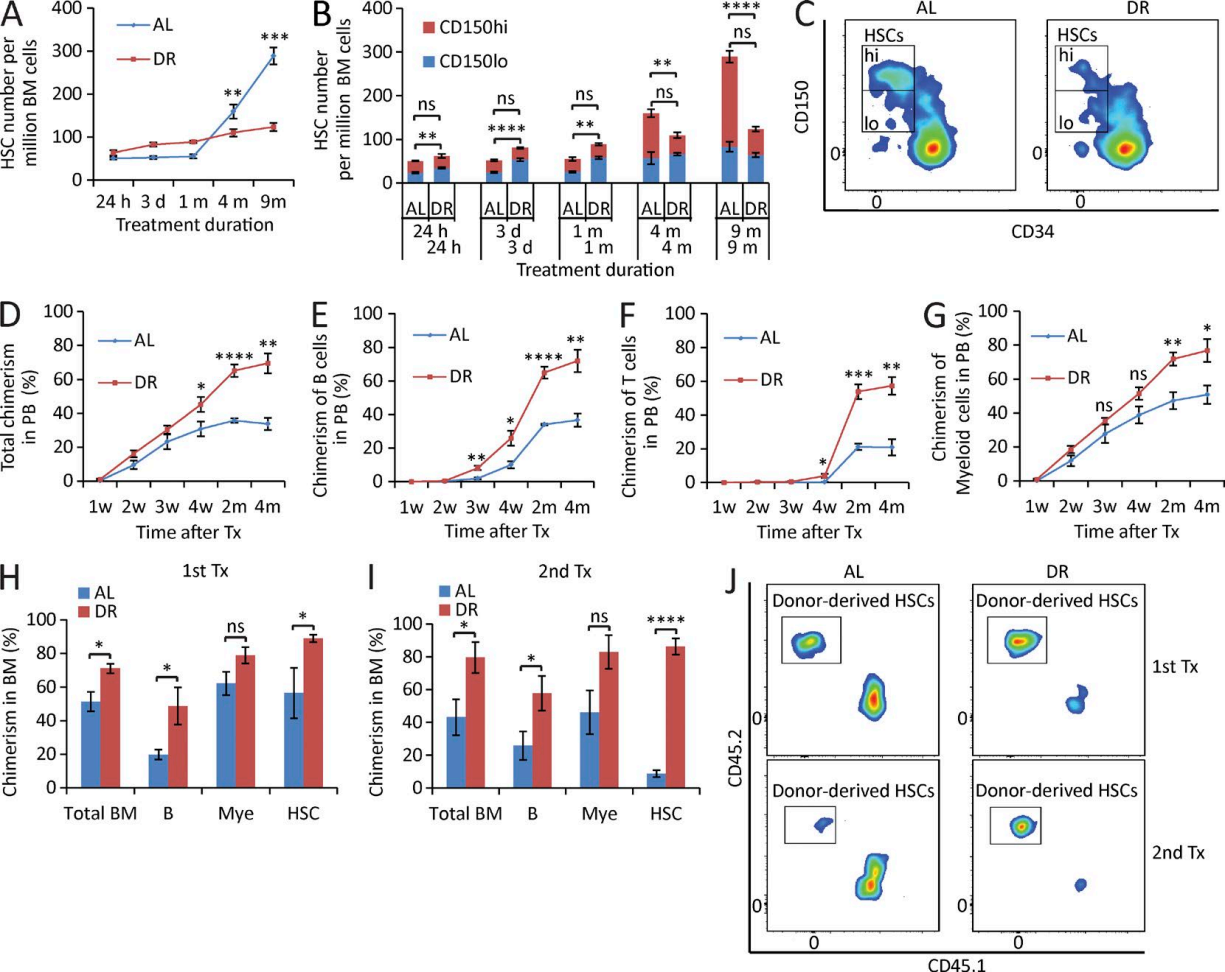
**DR retards HSC aging.** (A–C) 3-mo-old mice were fed with DR diet or AL diet. The HSC population was analyzed by flow cytometry at the indicated time points after dietary intervention (*n* = 3–5 mice per group per time point; *n* = 2 independent experiments). Note that the number of HSCs, in particular myeloid-biased HSCs, of DR mice was maintained relatively stable, whereas it increased significantly over time during aging in AL mice. In B, the significance of the comparison was shown in the lower line for the lymphoid-biased HSCs (CD150^lo^ HSCs) and in the upper line for the myeloid-biased HSCs (CD150^hi^ HSCs). Note that the skewing toward myeloid-biased HSCs during aging in AL mice was rescued in DR mice. (C) Representative FACS plots of mice treated with 9-mo DR or AL gated from c-Kit^+^Sca-1^+^lineage^−^ BM cells. (D–G) 100 HSCs derived from donor mice treated with mid-term (6 mo) DR or AL were transplanted along with 2 × 10^5^ total BM cells from competitor mice into recipient mice (*n* = 4–5 mice per group; *n* = 2 independent experiments). Panels show donor-derived total chimerisms (D), chimerisms of lymphoid lineage (E and F), and chimerisms of myeloid lineage (G) in PB at the indicated time points after transplantation. (H–J) 200 HSCs derived from donor mice treated with long-term (1 yr) DR or AL were transplanted along with 2 × 10^5^ total BM cells from competitor mice into recipient mice. 4 mo later, 10^7^ BM cells from the primary recipients were transplanted to secondary recipient mice (*n* = 4–5 mice per group; *n* = 2 independent experiments). (H and I) Donor-derived chimerisms in BM 4 mo after primary (H) and secondary (I) transplantation (Tx). (J) Representative FACS plots of primary and secondary recipient mice gated from HSCs. HSCs, CD150^+^CD34^−^c-Kit^+^Sca-1^+^lineage^−^ BM cells; m, months; B, B cells; T, T cells; Mye, myeloid cells; w, weeks. Data are displayed as mean ± SEM. *, P < 0.05; **, P < 0.01; ***, P < 0.001; ****, P < 0.0001 by unpaired two-tailed Student’s t test. ns, not significant.

